# Whole-genome resequencing reveals genetic diversity, differentiation, and selection signatures of yak breeds/populations in southwestern China

**DOI:** 10.3389/fgene.2024.1382128

**Published:** 2024-05-30

**Authors:** Shilin Zhang, Jing Li, Yanhua Zhao, Yujun Tang, Hao Li, Tianzeng Song, Tianwu An, Jiuqiang Guan, Xiaowei Li, Ming Zhang

**Affiliations:** ^1^ College of Animal Science and Technology, Sichuan Agricultural University, Chengdu, China; ^2^ Institute of Animal Science, Tibet Academy of Agricultural and Animal Husbandry Science, Lhasa, China; ^3^ Sichuan Academy of Grassland Science, Chengdu, China; ^4^ Breeding Fram of Longri, Agriculture and Rural Bureau of Aba Prefecture in Sichuan, Hongyuan, China; ^5^ Key Laboratory of Livestock and Poultry Multi-Omics, Ministry of Agriculture and Rural Affairs, College of Animal Science and Technology, Sichuan Agricultural University, Chengdu, China; ^6^ Farm Animal Genetic Resources Exploration and Innovation Key Laboratory of Sichuan Province, College of Animal Science and Technology, Sichuan Agricultural University, Chengdu, China

**Keywords:** Bos grunniens, whole-genome resequencing, genomic diversity, population genetic structure, selection signature

## Abstract

The Sichuan-Yunnan region is the main production area of yaks in southwestern China, with rich genetic resources of Yaks. Nevertheless, there have been limited study on the genetic characteristics of the entire yak populations in Tibet and southwestern China. In this study, we performed whole-genome resequencing to identify genetic variation information in a total of 198 individuals from six yak breeds (populations) in Sichuan (Muli yak, Jinchuan yak, Changtai yak, Maiwa yak), Yunnan (Zhongdian yak), and Tibet (Tibetan yak). The aim was to investigate the whole-genome genetic diversity, population genetic structure, and genome selection signatures. We observed that all six populations exhibit abundant genetic diversity. Except for Tibetan yaks, which showed low nucleotide diversity (0.00104), the remaining yak populations generally displayed high nucleotide diversity (0.00129–0.00153). Population genetic structure analysis revealed that, among the six yak populations, Muli yak exhibited greater differentiation from other yak populations and formed a distinct cluster independently. The Maiwa yak population displayed a complex genetic structure and exhibited gene exchange with Jinchuan and Changtai yaks. Positive selection signals were detected in candidate genes associated with growth (*GNB4*, *HMGA2*, *TRPS1*, and *LTBP1*), reproduction (*PI4KB*, *DYNC1I1*, and *GRIP1*), immunity (*CD200* and *IL1RAP*), lactation (*SNX13* and *CPM*), hypoxia adaptation (*NDUFB6*, *PRKN*, and *MRPS9*), hair (*KRT24*, *KRT25*, and *KRT26*), meat quality (*SUCLG2*), digestion and absorption (*CLDN1*), and pigment deposition (*OCA2*) using the integrated Pi and *F*_ST_ methods. This study provides significant insights into understanding the whole-genome genetic characteristics of yak populations in Tibet and southwestern China.

## Introduction

Yaks are a unique livestock species in the plateau region, displaying high-altitude adaptation to extreme and harsh environments, including cold and hypoxia (Wiener et al., 2003; Aguiar et al., 2018; Qi et al., 2019). They hold significant importance in local animal husbandry, providing meat, milk, wool, labor, fuel, and other essential resources to the local herdsmen (Wiener et al., 2003). Additionally, yaks serve as a valuable gene bank for genetic studies (Qiu et al., 2012).

The exploration of population genetic structure of yak has become a prominent field in domestic animal. Studies have been conducted on the genetic diversity of yaks using microsatellites and mitochondrial DNA (Qiu et al., 2015; Wang et al., 2021; Hameed et al., 2022; Ma et al., 2022). Moreover, the assembly and publication of the first yak reference genome in 2012, followed by subsequent improvements, have resulted in a highly complete and accurate yak reference genome at the chromosome level (Qiu et al., 2012; Ji et al., 2021; Zhang et al., 2021). This achievement has laid a robust foundation for investigating genetic variation at the whole-genome level in yaks, leading to the widespread use of whole-genome resequencing technology. This technology has enabled analyses of genetic variation in yaks, covering aspects such as their origin, domestication (Medugorac et al., 2017; Chai et al., 2020; Meng et al., 2022), genetic diversity and structure (Wang et al., 2014; Zhang et al., 2016; Lan et al., 2018; Wang et al., 2019; Li et al., 2022), high-altitude adaptation (Qiu et al., 2012; Qiu et al., 2015; Guang Xin et al., 2019b; Lan et al., 2021; Gao et al., 2022) and selection pressures (Lan et al., 2018; Xie et al., 2018; Guang Xin et al., 2019a; Li et al., 2022).

Southwestern China, specifically Sichuan Province’s Garze Tibetan Autonomous Prefecture, Aba Tibetan and Qiang Autonomous Prefecture, and Yunnan Province’s Diqing Tibetan Autonomous Prefecture, serves as the primary production area for yaks (Lan et al., 2018). In this region, several yak breeds, including Jiulong yak, Maiwa yak, Muli yak, Jinchuan yak, Changtai yak, and Zhongdian yak, have been officially recognized as excellent indigenous breeds by the government (Lan et al., 2021). Each of these yak breeds possesses unique characteristics and outstanding traits. For example, the Jinchuan yak stands out with approximately 52% of individuals having 15 thoracic vertebrae and 15 pairs of ribs, one more thoracic vertebra and one more pair of ribs than other yak breeds (Lan et al., 2018). Additionally, multi-ribbed Jinchuan yaks demonstrate higher meat and milk production, as well as superior reproductive performance compared to other breeds (Mipam et al., 2012). Maiwa yaks, on the other hand, produce milk with a protein content 40%–60% higher than that of native bovine milk (Chen et al., 2021). Protecting, developing, and utilizing the genetic resources of yak populations in this region holds great significance for the advancement of China’s yak industry. However, comprehensive studies on genetic variation detection, and selection signal analysis at the whole-genome level for multiple yak breeds in this region remain unreported.

To address the genetic gap, this study detected genetic variation, elucidate the genetic diversity, explore population genetic structure and genome-wide selection using six yak populations including four from Sichuan (Maiwa yak, Muli yak, Jinchuan yak, and Changtai yak), one from Yunnan (Zhongdian yak), and a collection of Tibetan yak populations. Our findings provide a scientific and theoretical basis for the protection, development, and genetic improvement of yak genetic resources in Tibet and southwestern China.

## Materials and methods

### Sample collection and whole-genome resequencing

In this study, we collected a total of 69 blood samples from Maiwa yak (MW) (The altitude of each sampling location ranges from 3400 m to 3600 m), 20 from Zhongdian yak (ZD), and 59 from Tibetan yak (XZ). Additionally, we collected 18 tissue samples each from Muli yak (ML) and Changtai yak (CT), and 14 tissue samples from Jinchuan yak (JC). The Tibetan yak population includes 16 Niangya yaks (NY), 11 Chawula yaks (CWL), 6 Sibu yaks (SB), 17 Dangxiong yaks (DX), 3 Naqu yaks (NQ), and 6 Pali yaks (PL) (Supplementary Figure S1; Supplementary Table S1). Genomic DNA was extracted using the TIANamp Genomic DNA Kit (Tiangen Biotech Co., Ltd., Beijing, China). 59 qualified Tibetan yak genomic DNA samples were sent to Biomarker Technologies (Beijing, China) for paired-end sequencing on the Illumina HiSeq 2500 platform. The remaining yak genomic DNA samples were sent to BGI Genomics (Shenzhen, China) for paired-end sequencing on the T7 platform, with each read length of 150 bp.

### Read mapping and SNP calling

The raw data were filtered through the quality control process conducted by fastp 0.20.0 (Chen et al., 2018). The high-quality clean reads were mapped to the latest yak reference genome (BosGru3.0, GCA_005887515.2) using Burrows–Wheeler Aligner (BWA, v0.7.8-r455) software and SAMtools (Li and Durbin, 2009) to get the original mapping results stored in BAM format. Then, the results were dislodged duplication by Picard (http://broadinstitute.github.io/picard/). After genome mapping we undertook SNP calling for all individuals using SamTools, v1.13, and The “mpileup” command was executed to identify SNPs with the parameters as -m 2 -F 0.002 -d 1000 -u -C 50 (Li et al., 2015). The genotype data were filtered using PLINK 1.9 based on minor allele frequencies, missing genotype rates, and biallelic alleles with parameter: -geno 0.2 --maf 0.05 --biallelic-only. SnpEff (Cingolani et al., 2012) was used for functional annotation of variants. The variation data reported in this paper have been deposited in the Genome Variation Map (GVM) (Li et al., 2021) in National Genomics Data Center, Beijing Institute of Genomics, Chinese Academy of Sciences and China National Center for Bioinformation (CNCB-NGDC Members and Partners, 2022), under accession number GVM000675 (https://bigd.big.ac.cn/gvm/getProjectDetail?Project=GVM000675).

### Population genetic diversity analysis

We estimated the genomic nucleotide diversity (Pi) of each yak population using VCFtools (v0.1.16) (Danecek et al., 2011) with the parameters (-window-pi 50,000 -window-pi-step 20,000). VCFtools was also used to calculate the average observed heterozygosity (*H*_*O*_) and expected heterozygosity (*H*_*E*_) as well as the average inbreeding coefficient (*F*) for six yak populations using the -het parameter. The pattern of linkage disequilibrium (LD) among 198 yaks from six populations can be effectively reflected by calculating the coefficient of determination (*r* ^2^) between pairwise SNPs using PopLDdecay (Zhang et al., 2019) with default parameter.

### Population genetic structure and phylogenetic tree analysis

Linkage sites in the genomic data were removed with parameters (-indep-pair-wise 50 10 0.2) using PLINK (v1.9) software. The genetic structure was analyzed using ADMIXTURE (v1.3.0) and the optimal clustering was also calculated to determine the best K value using ADMIXTURE’s cross-validation procedure. The smartpca module in EIGENSOFT (Patterson et al., 2006) was used to perform principal component analysis (PCA) on six yaks populations. The results were plotted using the ggplot2 package in R for the first principal component (PC1) and the second principal component (PC2). Based on the pairwise distance matrix among samples, a neighbor-joining (NJ) tree was constructed by MEGA (v11.0.11). The original tree file was visualized and beautified using the ggtree package in R. VCFtools was used to calculate the fixation index (*F*_ST_) between six yak populations.

### Genome-wide selective sweep and functional enrichment analysis

Based on the results of *F*_ST_, we used the Muli yak as the control group and the Maiwa yak as the selection group to perform selection sweep based on two analysis methods, nucleotide diversity (Pi), and the genome-wide distribution of pairwise fixation index (*F*_ST_). Pi and *F*_ST_ was calculated with 50 kb sliding windows and 20 kb steps between adjacent windows using the VCFtools. The windows with the top 5% *F*_ST_ and -log10 Pi values were considered as candidate region under strong selection and the candidate genes in the regions were identified. The intersection of two analysis results was taken as the final selected genes and a Venn diagram was drawn using the online website jvenn (http://jvenn.toulouse.inra.fr/app/index.html). The functions of the selected genes were further explored by conducting the Gene Ontology (GO) and Kyoto Encyclopedia of Genes and Genomes (KEGG) pathway enrichment analyses using KOBAS 3.0 (http://kobas.cbi.pku.edu.cn/anno_iden.php).

## Results

### Genome resequencing and genetic variation

Sequencing of 59 Tibetan yaks yielded 967.591 Gb of raw data and 953.379 Gb of filtered clean data. An additional 139 yaks were sequenced, producing 5274.237 Gb of raw data and 5084.898 Gb of filtered clean data (Supplementary Table S2). After mapping to the yak reference genome (BosGru3.0), the sequencing depth of the 59 individual Tibetan yaks ranged from 3.45 to 4.31×, while the sequencing depth of the remaining 139 yaks ranged from 9.12 to 25.42× (Supplementary Table S3). The mapping rate for all samples was above 96.68%. In total, we obtained 44,262,798 high-quality SNPs. The highest number of SNPs (5,145,209) was detected in Tibetan yaks, followed by Changtai yaks (3,007,140), Jinchuan yaks (2,955,926), Muli yaks (2,951,598), and Maiwa yaks (2,764,492), while the lowest number of SNPs was detected in Zhongdian yaks (2,640,551). Most SNPs were distributed in intergenic and intronic regions, with 63.90% located in intergenic regions and 33.84% in intronic regions. Only 0.61% of SNPs were distributed in exonic regions. Of the SNPs in exonic regions, 39.62% were synonymous and 36.53% were non-synonymous (Table 1).

**TABLE 1:** SNP functional annotation results.

Category		Number of SNPs	Proportion (%)
**Upstream**		29816	0.453
**UTR3**		33663	0.512
**UTR5**		7299	0.111
**Exonic**	Stop gain	270	0.004
	Stop loss	58	0.001
	Synonymous	15846	0.241
	Non-synonymous	14609	0.222
	Unknown	9211	0.140
**Intronic**		2226657	33.843
**Splicing**		334	0.005
**Downstream**		36667	0.557
**Upstream/downstream**		1045	0.016
**Intergenic**		4203881	63.895

### Population genomic diversity

**FIGURE 1:**
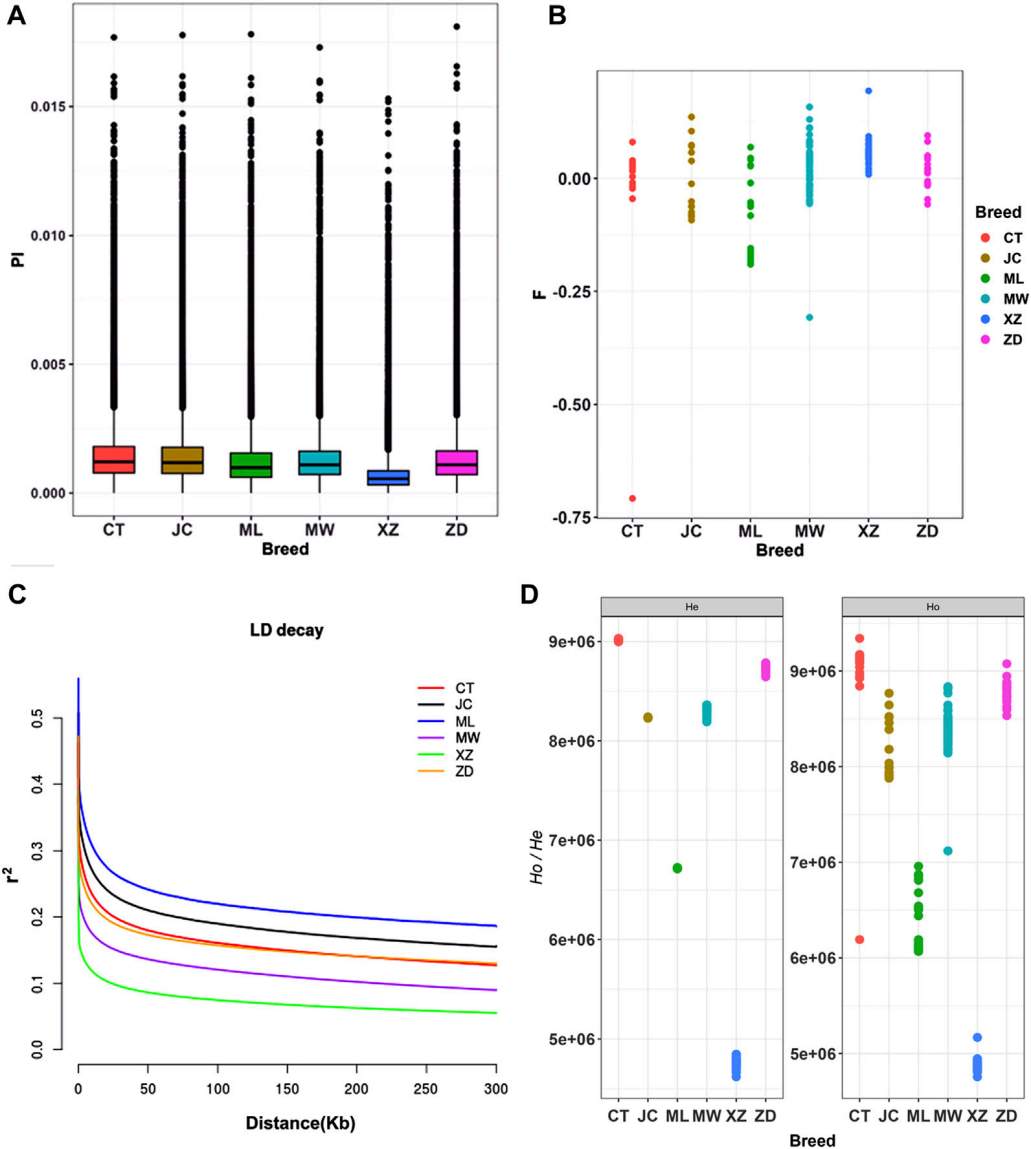
Analysis of genomic diversity, Pi, *F* and LD for these yak breeds/populations in southwestern China. **(A)** Box plots of nucleotide diversity (Pi). **(B)** the average inbreeding coefficient (*F*). **(C)** Decay of linkage disequilibrium (LD). **(D)** The observed heterozygosity (*Ho*) and expected heterozygosity (*He*) CT: Changtai yak, JC: Jinchuan yak, ML:Muli yak, MW: Maiwa yak, XZ: Tibet yak, ZD: Zhongdian yak.

Pi among the six breeds of yaks ranged from 0.00104 to 0.00153 (Figure 1A). Changtai yak exhibited the highest Pi at 0.00153, while Tibetan yak had the lowest at 0.00104. Apart from Tibetan yak, which showed relatively low Pi, the other yak populations generally exhibited high Pi, ranging from 0.00129 to 0.00153, (Supplementary Table S4). Except for Tibetan yak (0.05707), the *F* of the other five yak breeds was relatively low (−0.07969 to 0.01778) (Supplementary Table S4, Figure 1B). Muli yak had the lowest average inbreeding coefficient at −0.07969. The LD analysis showed that Muli yak had the highest average LD (*r* ^2^), followed by Jinchuan yak, Changtai yak, Zhongdian yak, and Maiwa yak. In contrast, Tibetan yaks had the lowest average LD (*r* ^2^) (Figure 1C). Results for heterozygosity indicated that the differences between observed heterozygosity (*Ho*) and expected heterozygosity (*He*) among the six breeds of yaks were small (Figure 1D). Muli yak had the highest *Ho* and *He*, at 0.36546 and 0.33848, respectively, while Tibetan yak had the lowest *Ho* and *He*, at 0.27363 and 0.29020, respectively (Supplementary Table S4). This suggests that Tibetan yak had the fastest LD decay rate among these six breeds, while Muli yak had the slowest decay rate.

### Population genetic structure

**FIGURE 2:**
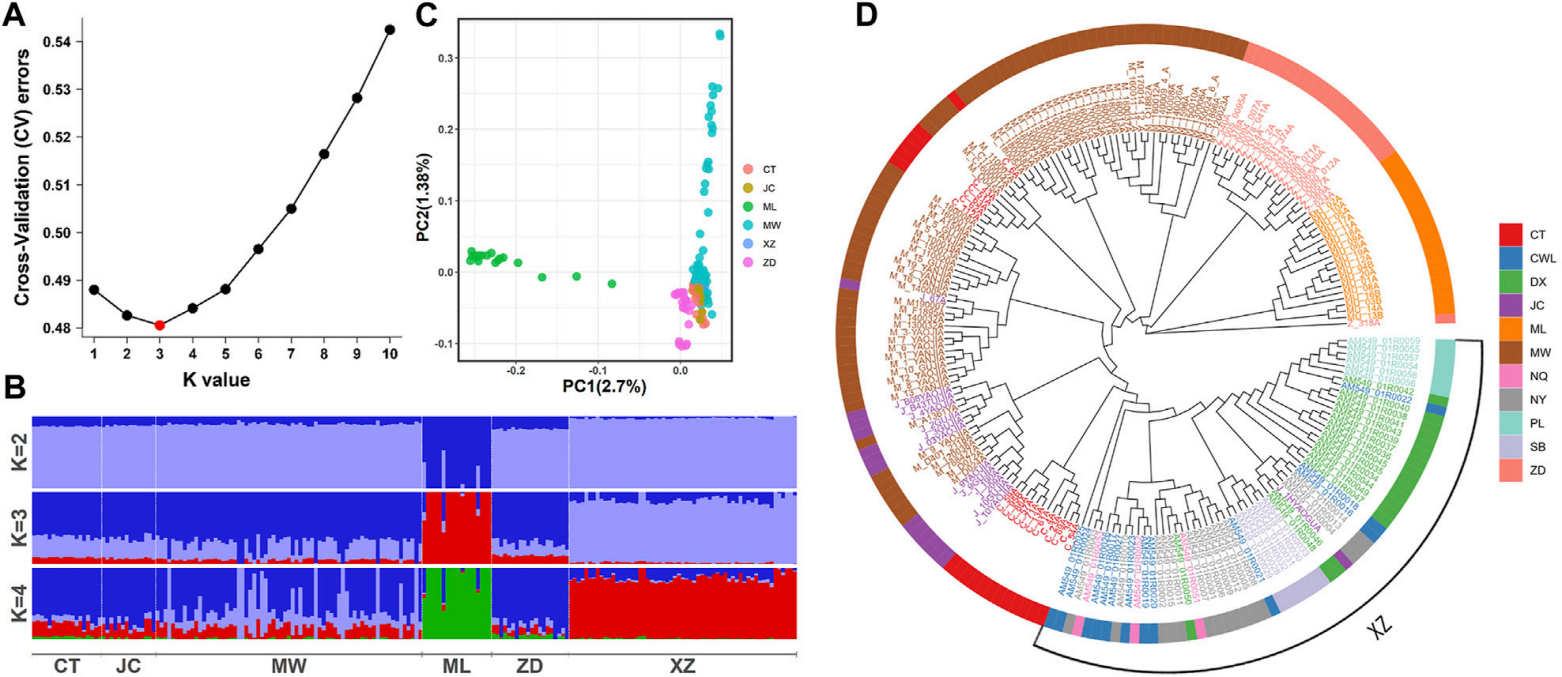
Population genetic structure of six yak breeds/population. **(A)** Cross-validation (CV) errors. **(B)** Analysis of ancestor components. **(C)** Principal components analysis. **(D)** Phylogenetic tree construction by neighbor-joining method. CT: Changtai yak. JC: Jinchuan yak. ML:Muli yak. MW: Maiwa yak. ZD: Zhongdian yak. XZ: Tibet yak, and it includes CWL, NQ, DX, NY, SB and PL poputation.

To examine the population genetic structure and relationships among six different yak breeds, we conducted a series of genome-wide analyses, including population genetic structure, PCA, and phylogenetic analysis. The results of the ancestral component analysis showed that the K value was equal to 3 and the lowest value of the cross-validation (CV) error was reached, proving that the optimal population grouping was when the ancestor population was 3 (Figure 2A). Moreover, when K = 2, the six yak breeds were grouped into two ancestral populations, distinguishing Muli yak from the other yak breeds. When K = 3, a new ancestral population emerged, further distinguishing Muli yak, Tibetan yak, and the other yak breeds. Most Muli yak samples have a relatively homogeneous ancestral population, while Tibetan yak showed a small number of mixed third ancestry. The majority of the other yak breeds showed a composition of three ancestries. When K = 4, the overall ancestry composition of each breed remained consistent with the results at K = 3 (Figure 2B). However, there was an uneven distribution phenomenon in the ancestry composition ratio of each sample of Maiwa yak.

According to the PCA results (Figure 2C), we observed that the Muli yak population formed a distinct and separate cluster from the other yak populations. On the other hand, most Maiwa yaks overlapped with the Jinchuan yaks, Changtai yaks, Tibetan yaks, and Zhongdian yaks, forming a clustered group together.

Based on genome-wide data, the NJ tree revealed that Muli yaks and Zhongdian yaks were grouped into two distinct branches, with Maiwa yaks forming a separate branch (Figure 2D). Some individuals from the Changtai yak and Jinchuan yak populations clustered together within the Maiwa yak branch, indicating close genetic relatedness. Similarly, some individuals from the Changtai yak and Tibetan yak populations formed another branch within the Maiwa yak group, suggesting a close genetic relationship between them. Notably, one individual from the Zhongdian yak population exhibited significant hybridization and formed a separated branch from the main population to form its own branch. Furthermore, one individual from the Jinchuan yak population clustered with the Tibetan yak population. Within the Tibetan yak population, the Pali yaks displayed a relatively pure bloodline, reflecting their distant geographic distribution from other Tibetan yaks.

The average *F*_ST_ of Muli yak was found to be the highest at 0.0898 (Table 2), indicating a moderate degree of genetic differentiation between Muli yak and other yak populations. On the other hand, the average fixation index of other yak populations was relatively low, with Maiwa yak having the lowest average differentiation index at 0.0345.

**TABLE 2:** *F*_ST_ values between pairwise populations.

Breeds/Population	JC	ML	MW	XZ	ZD	Mean
**Changtai (CT)**	0.0342	0.0868	0.0204	0.0315	0.0317	0.0409
**Jinchuan (JC)**		0.0999	0.0286	0.0422	0.0444	0.0499
**Muli (ML)**			0.0777	0.0998	0.0848	0.0898
**Maiwa (MW)**				0.0216	0.0241	0.0345
**Tibet (XZ)**					0.0371	0.0464
**Zhongdian (ZD)**						0.0444

### Genome-wide selective sweeps and functional enrichment analysis of candidate genes

**FIGURE 3:**
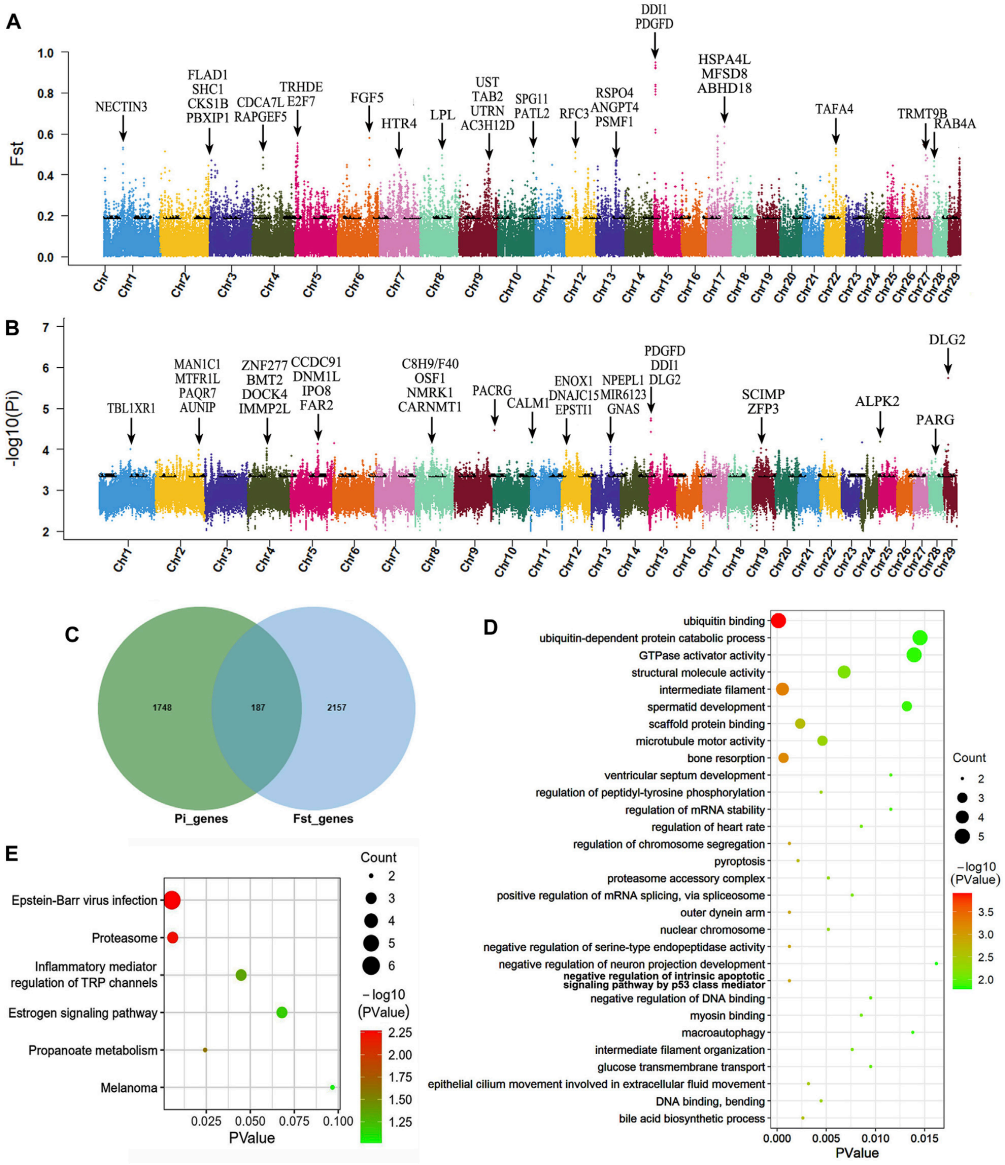
Genome-wide selective sweeps and functional analysis of Maiwa yak. Manhattan map based on *F*_ST_ **(A)** and Pi **(B)** selected detection methods. **(C)** Venn diagram based on two selected detection methods. **(D)** Top-30 GO items. **(E)** KEGG enrichment analysis of candidate genes for strong selection. Black arrow indicates candidate genes for strong selection in Figures 3A, B.

According to the results of genetic differentiation between populations, we observed that the average *F*_ST_ between Muli and Maiwa yak was the highest, indicating a moderate level of differentiation. Consequently, we designated Muli yak as the control group and Maiwa yak as the selection group for the selection sweep analysis using two methods, Pi and *F*_ST_. Subsequently, we identied the top 5% of scanning results as candidate regions (Figures 3A, B). As a result, a total of 1935 genes (Pi) and 2344 genes (*F*_ST_) were identified, with 187 genes overlapping between the two analyses (Figure 3C). These candidate genes were subject to strong selection and mainly participated in biological processes such as growth (*GNB4*, *HMGA2*, *TRPS1*, and *LTBP1*), reproduction (*PI4KB*, *DYNC1I1*, and *GRIP1*), immunity (*CD200* and *IL1RAP*), neural development (*ST3GAL3*, *KIAA0319L*, and *SFPQ*), lipid metabolism and accumulation (*CYP27A1*, *AGMO*, and *KBTBD2*), lactation (*SNX13* and *CPM*), bone development (*ZNF687* and *PPP2R2B*), hypoxia adaptation (*NDUFB6*, *PRKN*, and *MRPS9*), hair development (*KRT24*, *KRT25*, and *KRT26*), meat quality (*SUCLG2*), digestion and absorption (*CLDN1*), and pigment deposition (*OCA2*).

Functional enrichment analysis using Gene Ontology (GO) was performed on candidate genes, revealing GO terms with a significance level of *p* < 0.05 (Figure 3D). The analysis showed that The 187 selected genes were predominantly enriched in functions associated with immune regulation, reproductive regulation, regulation of heart rate, cell apoptosis, mitosis, digestive synthesis and metabolism, transmembrane substance transport, epithelial cell proliferation and wound healing, hormone secretion and regulation, bone resorption, heme transport, and ventricular septum development. Furthermore, KEGG pathway enrichment analysis was performed, identifying pathways with a significance level of *p* < 0.1 (Figure 3E). The identified pathways were linked to virus infection, propanoate metabolism, proteasome, inflammatory mediator regulation of TRP channels, estrogen signaling, and melanoma.

## Discussion

Genetic diversity reflects the adaptability of a population to its environment and its evolutionary history. It holds significant value for the protection and further utilization of animal genetic resources. In this study, we observed that the nucleotide diversity of Tibetan yak is low, likely due to their isolated geographical location, leading to limited genetic interchange with other populations. Chai et al. (2020) classeified 91 domestic yaks from 31 populations into three groups based on their phylogenetic relationships.They noted that the nucleotide diversity and genetic diversity of the yak population in the central Tibetan region were both low, aligning with our study results.

The observed heterozygosity (Ho) of Muli yak, Jinchuan yak, and Changtai yak was higher than the expected heterozygosity, and the inbreeding coefficient was generally lower. This suggests that these populations abundant exhibit genetic diversity and a low degree of inbreeding. This may be attributed to the large-scale purebred breeding and hybrid improvement efforts carried out on yaks in Sichuan in recent years. As a result of this work, several yak varieties have been developed for breeding populations and core breeding groups. On the other hand, the observed heterozygosity of Tibetan yak was low, while the inbreeding coefficient was high. This suggests a lack of genetic diversity, possibly due to the traditional feeding practices in the region that have led to significant inbreeding and a decline in reproductive performance. The LD decay analysis results indicated that Muli yak, Jinchuan yak, and Changtai yak have experienced more intense artificial selection in recent years, as evidenced by their slower LD decay rate. as evidenced by their slower LD decay rate. In contrast, Tibetan yak exhibited a faster LD decay compared to other populations, indicating less human intervention. This aligns with previous LD decay analysis results for Jinchuan yak and Tibetan yak (Wang et al., 2020).

In previous studies, Lan et al. (2018) investigated the population genetic structure of Jinchuan yaks at the whole-genome level. The results from phylogenetic tree and PCA analysis both indicated that Jinchuan yaks form an independent branch within the domestic yak population and are distantly related to Zhongdian yak, Tibetan yak, and Qinghai yak. Additionally, the ancestral component analysis results revealed that when K = 3, the Jinchuan yak population was separated from the rest of the domestic yak population. In the present study, the ancestral component analysis, PCA analysis, and *F*_ST_ statistics results of the six yak populations all pointed towards Muli yak exhibiting a relatively independent population genetic structure and distant relationship with other yak populations. Most of the Maiwa yak showed a high degree of hybridization, characterized by the smallest average differentiation index and a complex genetic background. This genetic variation is likely influenced by the diverse geographical environment of the two primary yak breeding regions. Muli yak and Maiwa yak are classified as the “Hengduan Mountains type” and “Qinghai-Tibet Plateau type,” respectively, based on their ecological characteristics. Muli yak predominantly inhabits mountain valleys, leading to grassland fragmentation and restricted gene flow, resulting in isolated breeding populations with. artificial selection. Conversely, Maiwa yak is primarily found in high-altitude terrains with espansive grasslands, where primitive year-round grazing is common, leading to a lower degree of artificial selection. It is speculated that the Maiwa yak population in this study may have originated from two distinct large populations due to the establishment of a core breeding group for Maiwa yak and the implementation of various effective breeding practices. One population may have introduced Jinchuan and Changtai yak bloodlines, while the other population retained a relatively pure genetic lineage without external influences.

The identification of selective signatures in a population is crucial for understanding its evolutionary history and economic traits. A total of 187 candidate positively selected genes were identified in the Maiwa yak population, and their potential effects on relevant important traits were discussed. For example, the *CLDN1* gene, a component of endothelial tight junctions, has been demonstrated to decrease intestinal permeability and improve immune adaptability in calves through the upregulation of its expression (Ghaffari et al., 2021). It also plays an effective role in repairing the rumen epithelial barrier of slow-growing yaks (Hu et al., 2019). Thus, the *CLDN1* gene may be associated with the stronger gastrointestinal immune function of Maiwa yaks, enabling them to adapt to the challenging high-altitude wetland grassland environment. The *GNB4* gene can control cattle growth by influencing the expression of growth-related hormones in the pituitary gland (Lu et al., 2020). The *HMGA2* gene, a transcription factor of insulin-like growth factor gene 2 (IGF2), plays a significant role in the growth trait of navel length in beef cattle (Aguiar et al., 2018). Similarly, the *TRPS1* gene is significantly associated with the increase in both cattle body weight and eye muscle area (Lee et al., 2011). Therefore, the *GNB4*, *HMGA2*, and *TRPS1* genes are likely to be closely associated with the growth and development of Maiwa yak.

This study also identified several genes that are specifically associated with reproduction. For instance, *PI4KB* is responsible for regulating actin aggregation during sperm capacitation (Etkovitz et al., 2007). *DYNC1I1* participates in transporting molecules and organelles during oocyte maturation and is the most prevalent transport system in cells (Racedo et al., 2008). *GRIP1* has been validated as a marker for detecting cow estrus (Hlaing et al., 2001; Lee et al., 2017). *CARPT* is a novel regulatory factor for ovarian function during follicular wave emergence in cows and is thought to play a potential role in dominant follicle selection during the follicular wave period in single-ovulating species as an “ovulation quota gatekeeper” (Mihm et al., 2000; Lv et al., 2009; Smith et al., 2010). These genes may have an impact on maintaining a stable reproductive pattern of Maiwa yak in the harsh high-altitude environments.

The challenging high-altitude environment and extensive productionpractices have resulted in the strong selection of genes associated with immunity and adaptation to hypoxia in the genome of Maiwa yak. For example, the reduction of the *CD200* gene has a significantly affects on the quantity of pulmonary epithelial cells in radiation conditions (Beach et al., 2023). This finding might elucidate the relevant mechanism that enables the Maiwa yak to adapt to high-altitude with intense radiation and maintain lung function without harm. The *IL1RAP* gene has been identified as a central gene encoding the interleukin-1 receptor accessory protein, which plays a significant role in the immune system (Dos Santos Silva et al., 2019). Furthermore, three genes, *NDUFB6*, *PRKN*, and *MRPS9*, are closely linked to mitochondrial structure and normal function (Buggiotti et al., 2021; Fang et al., 2022; Zhang et al., 2022). Their influence on respiratory and energy metabolism efficiency may be be associated with Maiwa yak’s adaptation to high-altitude hypoxic environments.

In addition, this study identified lactation-related genes *SNX13* and *CPM* among the selected genes of Maiwa yak. The *SNX13* gene is highly correlated with milk production traits in cows, while the *CPM* gene has a significant effect on the fatty acid content in cow milk (Rincón et al., 2009; Shi et al., 2020). The *SUCLG2* gene has been identified as a candidate biomarker for beef tenderness in cattle (Li and Li, 2021).

Three keratin family genes, *KRT24*, *KRT25*, and *KRT26*, have been identified. The *KRT24* gene play a crucial role in hair growth and follicle development in yaks (Bao et al., 2022). The long and dense wool of Maiwa yak helps them retain heat and withstand cold weather at high altitudes. It is worth noting that a pigment deposition-related gene, Additionally, an *OCA2* gene related to pigment deposition has been identified, potentially influencing black pigment deposition in Maiwa yak’s wool (Grønskov et al., 2007; Caduff et al., 2017; Ballan et al., 2022).

## Conclusion

In conclusion, this study systematically investigates the genetic diversity, population genetic structure, kinship relationships, genetic differentiation, and gene selection of several yak breeds (populations) in Tibet and southwestern China at the whole-genome level. Overall, all six populations exhibit rich genetic diversity. Muli yak shows higher differentiation from other yak populations and forms an independent cluster. The Maiwa yak population demonstrates a complex genetic structure. The candidate genes identified for Maiwa yak are associated with various functions such as growth, reproduction, immunity, lactation, hypoxia adaptation, hair development, meat quality, digestion and absorption, and pigment deposition. These findings provide a scientific and theoretical foundation for the exploration and conservation of yak genetic resources in this region, as well as for the selection and breeding of yak breeds.

## Data Availability

The datasets presented in this study can be found in online repositories. The names of the repository/repositories and accession number(s) can be found below: https://bigd.big.ac.cn/gvm/getProjectDetail?Project&equals;GVM000675, GVM000675.
