# Supplementary material for: Whole-genome resequencing reveals genetic diversity, differentiation, and selection signatures of yak breeds/populations in southwestern China
Source: Front Genet. 2024 May 30;15:1382128. doi: 10.3389/fgene.2024.1382128 (PMC11169580; doi:10.3389/fgene.2024.1382128)
Supplement: Supplementary file 1 [file Table1.DOCX]

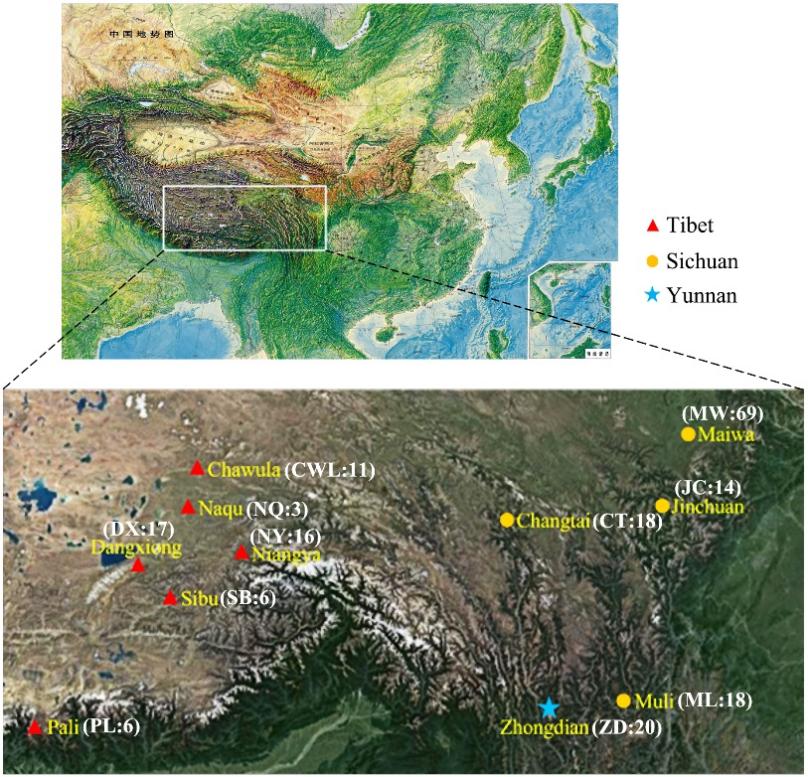


**Figure S1** The number and geographical distribution of yak samples from southwestern China in the study. The samples are from Tibet, Sichuan and Yunnan. In the bracket, (Abbreviation of breeds/population name: number of individuals)

**Table S1** Sample collection size and source of yak

|  | **Breed / population** | **Abbreviation** | **Sample size** | **Sample location** |
| --- | --- | --- | --- | --- |
| **1** | Maiwa yak | MW | 69 | Hongyuan County, Sichuan Province |
| **2** | Muli yak | ML | 18 | Muli County, Sichuan Province |
| **3** | Jinchuan yak | JC | 14 | Jinchuan County, Sichuan Province |
| **4** | Changtai yak | CT | 18 | Baiyu County, Sichuan Province |
| **5** | Zhongdian yak | ZD | 20 | Shangri La County, Yunnan Province |
| **6** | Nianya yak | NY | 16 | Lhari County, Tibet Autonomous Region |
| **7** | Chawula yak | CWL | 11 | Nyainrong County, Tibet Autonomous Region |
| **8** | Sibu yak | SB | 6 | Maizhokunggar County, Tibet Autonomous Region |
| **9** | Dangxiong yak | DX | 17 | Damxung County, Tibet Autonomous Region |
| **10** | Nagqu yak | NQ | 3 | Nagqu County, Tibet Autonomous Region |
| **11** | Pali yak | PL | 6 | Yadong County, Tibet Autonomous Region |
| **total** |  |  | 198 |  |

**Table S2** Summary of sequencing data quality

| Sample | Raw bases(bp) | Clean bases(bp) | Effective rate(%) | Error rate(%) | Q20(%) | Q30(%) | GC content(%) |
| --- | --- | --- | --- | --- | --- | --- | --- |
| C_112A | 43929625200 | 42779100000 | 97.38 | 0.03 | 97.33 | 91.65 | 43.03 |
| C_13A | 36065930700 | 35233830900 | 97.69 | 0.03 | 97.38 | 91.81 | 43.05 |
| C_23A | 33832726500 | 33151354500 | 97.99 | 0.03 | 96.97 | 90.54 | 43.08 |
| C_34 | 37256790300 | 36352904100 | 97.57 | 0.03 | 97.19 | 91.25 | 43.3 |
| C_43A | 59344977600 | 54752469300 | 92.26 | 0.03 | 97.99 | 93.7 | 44.19 |
| C_4A | 33703295400 | 32900876400 | 97.62 | 0.03 | 97.05 | 90.79 | 43.2 |
| C_51A | 27216384600 | 26690220600 | 98.07 | 0.03 | 96.97 | 90.57 | 43.27 |
| C_54A | 40708337700 | 39818122800 | 97.81 | 0.03 | 97.09 | 90.94 | 42.91 |
| C_57A | 32895424200 | 32195785500 | 97.87 | 0.03 | 96.72 | 89.85 | 42.77 |
| C_59A | 37326305100 | 36462333900 | 97.69 | 0.03 | 97.97 | 93.45 | 42.48 |
| C_61A | 37788224700 | 36914952000 | 97.69 | 0.03 | 97.17 | 91.19 | 43.16 |
| C_62A | 28520045400 | 27958141800 | 98.03 | 0.03 | 97.22 | 91.36 | 43.23 |
| C_63A | 35428798200 | 34632543600 | 97.75 | 0.03 | 97.3 | 91.56 | 43.16 |
| C_69A | 78816815400 | 77081494200 | 97.8 | 0.03 | 97.19 | 91.25 | 43.13 |
| C_6A | 36710739600 | 35990425500 | 98.04 | 0.03 | 96.94 | 90.47 | 42.87 |
| C_71A | 34371489000 | 33716011500 | 98.09 | 0.03 | 96.81 | 90.08 | 42.93 |
| C_86A | 34195305900 | 33458826000 | 97.85 | 0.03 | 97.02 | 90.7 | 42.99 |
| C_95B | 50499770700 | 49240600500 | 97.51 | 0.03 | 97.47 | 91.84 | 43.47 |
| J_03YAOJIA | 39853954800 | 38881409100 | 97.56 | 0.03 | 97.53 | 92.03 | 42.72 |
| J_10GUJIA | 37596341400 | 36663513000 | 97.52 | 0.03 | 97.31 | 91.32 | 42.65 |
| J_10YAOJIA | 35328679500 | 34375471800 | 97.3 | 0.03 | 96.75 | 89.59 | 42.73 |
| J_190A | 39685743600 | 38666205000 | 97.43 | 0.03 | 97.51 | 91.97 | 42.52 |
| J_1HYAOGUA | 39448385700 | 38450463900 | 97.47 | 0.03 | 97.42 | 91.69 | 42.72 |
| J_209A | 38940866700 | 38003434800 | 97.59 | 0.03 | 97.47 | 91.86 | 42.76 |
| J_2GUJIA | 39516804300 | 38487841800 | 97.4 | 0.03 | 97.5 | 91.97 | 43.01 |
| J_3GUJIA | 32224709100 | 31513794000 | 97.79 | 0.03 | 97.34 | 91.43 | 42.54 |
| J_4YAOJIA | 38331253500 | 37489918200 | 97.81 | 0.03 | 97.39 | 91.59 | 42.84 |
| J_67A | 38229570000 | 37199320800 | 97.31 | 0.03 | 97.31 | 91.33 | 42.62 |
| J_9GUJIA | 42536249700 | 41432349600 | 97.4 | 0.03 | 97.67 | 92.47 | 42.84 |
| J_9YAOJIA | 39337601700 | 38317231200 | 97.41 | 0.03 | 97.42 | 91.69 | 42.72 |
| J_B08YAOJIA | 39997212600 | 38887835400 | 97.23 | 0.03 | 97.63 | 92.36 | 42.96 |
| J_B43TUIJIA | 45375064200 | 44155668000 | 97.31 | 0.03 | 97.48 | 91.86 | 42.77 |
| M_100019A | 27861610200 | 26469840600 | 95 | 0.03 | 97.09 | 91.63 | 43.34 |
| M_100073A | 41612311500 | 40749771600 | 97.93 | 0.03 | 97.8 | 92.56 | 43.18 |
| M_10_GUJIA | 33210455100 | 32402964600 | 97.57 | 0.03 | 97.59 | 92.23 | 42.97 |
| M_110002A | 30523534800 | 29461259400 | 96.52 | 0.03 | 96.83 | 90.98 | 42.65 |
| M_110008A | 37828178100 | 36580246800 | 96.7 | 0.03 | 97.75 | 92.43 | 43.06 |
| M_110020A | 39028832700 | 37422000300 | 95.88 | 0.03 | 97.19 | 90.86 | 43.27 |
| M_11_YANJIA | 39760062600 | 38694109800 | 97.32 | 0.03 | 97.43 | 91.73 | 42.83 |
| M_120023A | 27147510600 | 26095975500 | 96.13 | 0.03 | 96.91 | 91.14 | 43.27 |
| M_120038A | 29844863400 | 28842037500 | 96.64 | 0.03 | 96.8 | 90.91 | 43.03 |
| M_12_YAOJIA | 41677601400 | 40564609800 | 97.33 | 0.03 | 97.62 | 92.34 | 42.82 |
| M_130006A | 27087136800 | 25966533000 | 95.86 | 0.03 | 96.79 | 90.89 | 43.09 |
| M_130032A | 42683623800 | 37857192900 | 88.69 | 0.03 | 98 | 93.47 | 43.52 |
| M_130033A | 38042864400 | 36826987500 | 96.8 | 0.03 | 98.12 | 93.7 | 43.36 |
| M_130034A | 36224292900 | 34753659900 | 95.94 | 0.02 | 98.13 | 93.97 | 42.98 |
| M_13_YANJIA | 50441798100 | 49188441900 | 97.52 | 0.03 | 97.51 | 91.97 | 42.83 |
| M_140005A | 27699969000 | 26822759700 | 96.83 | 0.03 | 96.98 | 91.32 | 43.06 |
| M_140007A | 39837875700 | 38345657700 | 96.25 | 0.03 | 97.85 | 92.81 | 43.41 |
| M_140009_4_A | 28216151100 | 27086489400 | 96 | 0.03 | 96.83 | 90.84 | 43.05 |
| M_140026A | 41173219500 | 39847728600 | 96.78 | 0.03 | 98 | 93.22 | 43.17 |
| M_140032A | 43241037300 | 41878859400 | 96.85 | 0.03 | 97.84 | 92.75 | 43.09 |
| M_140047A | 29269888800 | 28379457900 | 96.96 | 0.03 | 96.5 | 90.06 | 42.78 |
| M_140056A | 28530003000 | 27259773300 | 95.55 | 0.03 | 96.75 | 90.7 | 43.04 |
| M_150008A | 31586010300 | 30384886200 | 96.2 | 0.03 | 97.88 | 93.16 | 42.84 |
| M_150010A | 28140343200 | 27253925700 | 96.85 | 0.02 | 98.07 | 93.75 | 42.99 |
| M_150024_6_A | 32004741600 | 31109152500 | 97.2 | 0.03 | 97.92 | 93.31 | 42.73 |
| M_150073A | 47999881800 | 46279243500 | 96.42 | 0.03 | 98.03 | 93.66 | 42.91 |
| M_1596A | 32387224500 | 31255359600 | 96.51 | 0.03 | 96.84 | 91.02 | 43.05 |
| M_15_GUJIA | 39133903500 | 38142012300 | 97.47 | 0.03 | 97.56 | 92.14 | 42.86 |
| M_160001A | 40605419700 | 38807484000 | 95.57 | 0.02 | 98.12 | 93.97 | 42.83 |
| M_160006A | 40702570500 | 39363442200 | 96.71 | 0.03 | 97.93 | 93.04 | 43.17 |
| M_160011_11_A | 39516299100 | 37960649100 | 96.06 | 0.03 | 98.06 | 93.55 | 43.23 |
| M_160012A | 27383585100 | 26352328800 | 96.23 | 0.03 | 96.62 | 90.44 | 43.09 |
| M_160013A | 37692956100 | 36412662300 | 96.6 | 0.02 | 98.03 | 93.65 | 42.91 |
| M_160015A | 30439412700 | 29395021500 | 96.57 | 0.03 | 96.72 | 90.66 | 43.03 |
| M_160023A | 37686165600 | 36058029300 | 95.68 | 0.03 | 97.67 | 92.27 | 43.18 |
| M_160039A | 31482242400 | 30528874200 | 96.97 | 0.03 | 96.62 | 90.42 | 42.58 |
| M_16_YANJIA | 48222992100 | 46821850500 | 97.09 | 0.03 | 97.83 | 93 | 43.03 |
| M_170007A | 54516041700 | 44751304500 | 82.09 | 0.02 | 97.81 | 93.68 | 43.31 |
| M_170014_12_A | 26919351000 | 25696210500 | 95.46 | 0.03 | 96.96 | 91.15 | 43.09 |
| M_180001A | 41241626700 | 39161865900 | 94.96 | 0.02 | 98.17 | 94.09 | 43.09 |
| M_180036A | 32071590300 | 31045376700 | 96.8 | 0.03 | 97.93 | 93.34 | 42.79 |
| M_180037A | 37879183800 | 36567637200 | 96.54 | 0.03 | 98.03 | 93.64 | 42.75 |
| M_180054A | 31674974700 | 30679980900 | 96.86 | 0.03 | 96.62 | 90.39 | 43.12 |
| M_180058A | 43847942100 | 42123823800 | 96.07 | 0.03 | 98.05 | 93.47 | 43.41 |
| M_180073A | 37606370400 | 35804015400 | 95.21 | 0.03 | 97.87 | 92.91 | 43.45 |
| M_190063A | 37655743200 | 36399966600 | 96.67 | 0.03 | 97.95 | 93.1 | 43.07 |
| M_19_YANJIA | 43417701300 | 42282999000 | 97.39 | 0.03 | 97.53 | 92 | 42.9 |
| M_1_YANJIA | 42292879500 | 41244570600 | 97.52 | 0.03 | 97.67 | 92.5 | 42.94 |
| M_2_YANJIA | 35866642200 | 35007245400 | 97.6 | 0.03 | 96.72 | 89.78 | 42.86 |
| M_3-YAOJIA | 38143381200 | 37272348000 | 97.72 | 0.03 | 97.14 | 91.09 | 42.99 |
| M_51896A | 75915163200 | 73196353500 | 96.42 | 0.03 | 97.95 | 93.12 | 43.12 |
| M_5_GUJIA | 38367081600 | 37404643500 | 97.49 | 0.03 | 97.97 | 93.48 | 43.3 |
| M_5_YANJIA | 44256628500 | 43051659900 | 97.28 | 0.03 | 97.65 | 92.42 | 42.97 |
| M_6_YAOJIA | 38248053000 | 37295114700 | 97.51 | 0.03 | 97.52 | 91.97 | 42.62 |
| M_788427A | 37111321200 | 35595834000 | 95.92 | 0.03 | 97.91 | 93.02 | 43.3 |
| M_7_YAOJIA | 43924058400 | 42714770400 | 97.25 | 0.03 | 97.52 | 92 | 42.8 |
| M_8_YAOJIA | 36242081700 | 35341137000 | 97.51 | 0.03 | 97.44 | 91.75 | 42.81 |
| M_9_YAOJIA | 35289431400 | 34370183700 | 97.4 | 0.03 | 97.88 | 93.17 | 43.01 |
| M_A1361YA | 32941506900 | 31985815800 | 97.1 | 0.03 | 97.92 | 93 | 43.03 |
| M_A1508A | 36992705100 | 35529748200 | 96.05 | 0.02 | 98.09 | 93.86 | 43.01 |
| M_C160001A | 70156234500 | 61002249300 | 86.95 | 0.02 | 98.49 | 94.91 | 43.83 |
| M_C170004A | 28396128600 | 27414813000 | 96.54 | 0.03 | 96.87 | 91.08 | 42.99 |
| M_D024A | 39817522200 | 39023244900 | 98.01 | 0.03 | 97.56 | 92.15 | 43.41 |
| M_D045A | 35003885100 | 33608475000 | 96.01 | 0.03 | 97.91 | 93.12 | 43.06 |
| M_D072A | 42750405300 | 41572184100 | 97.24 | 0.03 | 97.78 | 92.53 | 43.22 |
| M_D401_2118A | 31240396500 | 30118476600 | 96.41 | 0.03 | 97.95 | 93.4 | 42.89 |
| M_F1895A | 40103556000 | 38916311700 | 97.04 | 0.03 | 97.69 | 92.26 | 43.16 |
| M_M190007 | 34468253400 | 33138049500 | 96.14 | 0.03 | 96.74 | 90.74 | 43.5 |
| M_Q1882A | 28423452300 | 27357495600 | 96.25 | 0.03 | 96.83 | 90.91 | 43.06 |
| MU_01A | 41531241300 | 40620636600 | 97.81 | 0.03 | 97.22 | 91.3 | 42.86 |
| MU_02A | 41409031500 | 40471121400 | 97.74 | 0.03 | 97.25 | 91.41 | 42.98 |
| MU_03B | 37765036200 | 36808077900 | 97.47 | 0.03 | 97.36 | 91.5 | 43.53 |
| MU_04B | 43366849200 | 42135472800 | 97.16 | 0.03 | 97.68 | 92.53 | 43.36 |
| MU_05A | 65733408600 | 54300711300 | 82.61 | 0.02 | 98.31 | 94.75 | 44.16 |
| MU_06A | 36748726800 | 35936089800 | 97.79 | 0.03 | 97.11 | 90.97 | 42.97 |
| MU_08A | 35456938800 | 34650466200 | 97.73 | 0.03 | 97.17 | 91.19 | 43.02 |
| MU_09A | 38849746200 | 38075807700 | 98.01 | 0.03 | 97.03 | 90.73 | 43 |
| MU_10A | 33721035000 | 32975261700 | 97.79 | 0.03 | 97.23 | 91.36 | 42.89 |
| MU_11A | 45050462700 | 44154016800 | 98.01 | 0.03 | 97.2 | 91.27 | 42.85 |
| MU_12A | 35387609400 | 34666339800 | 97.96 | 0.03 | 96.72 | 89.88 | 42.96 |
| MU_13B | 33905042400 | 33075472800 | 97.55 | 0.03 | 97.05 | 90.52 | 43.01 |
| MU_14A | 36653840100 | 35940130500 | 98.05 | 0.03 | 96.83 | 90.14 | 42.98 |
| MU_16B | 47376304500 | 46192059600 | 97.5 | 0.03 | 97.37 | 91.52 | 43.67 |
| MU_17A | 38007880800 | 37256793900 | 98.02 | 0.03 | 97.09 | 90.91 | 42.95 |
| MU_18A | 37818773700 | 37029689700 | 97.91 | 0.03 | 97.05 | 90.81 | 42.89 |
| MU_19A | 37550707200 | 36584422200 | 97.43 | 0.03 | 97.88 | 93.18 | 42.61 |
| MU_20A | 35163508200 | 34357402200 | 97.71 | 0.03 | 96.73 | 89.86 | 42.76 |
| Z_002A | 33445647600 | 32357025000 | 96.75 | 0.02 | 98.07 | 93.77 | 42.71 |
| Z_0057A | 36931912800 | 35705221800 | 96.68 | 0.02 | 98.09 | 93.84 | 42.89 |
| Z_005_0095A | 29276775900 | 28020466800 | 95.71 | 0.03 | 98.1 | 93.89 | 43.19 |
| Z_0065A | 31191944700 | 29851173000 | 95.7 | 0.03 | 97.6 | 92.35 | 42.93 |
| Z_0078_041A | 32697058800 | 31822560000 | 97.33 | 0.03 | 97.92 | 93.31 | 42.48 |
| Z_0080_012A | 30518670900 | 29631621000 | 97.09 | 0.03 | 97.44 | 91.75 | 43 |
| Z_0094_007A | 31716642900 | 30254122200 | 95.39 | 0.03 | 97.8 | 92.97 | 43.08 |
| Z_021A | 31955001000 | 30658755300 | 95.94 | 0.03 | 97.66 | 92.52 | 42.94 |
| Z_024A | 29376316200 | 28206844200 | 96.02 | 0.02 | 98.16 | 94.08 | 43.02 |
| Z_031_374A | 34564019700 | 33958542300 | 98.25 | 0.03 | 97.28 | 91.27 | 43.51 |
| Z_039_040A | 34408286100 | 32694843300 | 95.02 | 0.03 | 97.88 | 93.26 | 42.92 |
| Z_318A | 36381620100 | 34844028000 | 95.77 | 0.03 | 97.95 | 93.41 | 43.09 |
| Z_321A | 32104890600 | 30987160800 | 96.52 | 0.02 | 98.06 | 93.77 | 42.89 |
| Z_340A | 44389458900 | 43019582100 | 96.91 | 0.03 | 97.14 | 90.84 | 42.93 |
| Z_353_1A | 35339293200 | 34091708400 | 96.47 | 0.03 | 97.8 | 92.93 | 42.93 |
| Z_353_2A | 32606287800 | 31582948200 | 96.86 | 0.03 | 97.94 | 93.37 | 42.9 |
| Z_374A | 35478368700 | 33768307200 | 95.18 | 0.03 | 97.95 | 93.44 | 42.88 |
| Z_378A | 39134329800 | 37421053500 | 95.62 | 0.02 | 98.16 | 94.04 | 43.14 |
| Z_389A | 38041327200 | 36544512600 | 96.07 | 0.03 | 97.78 | 92.91 | 43.08 |
| Z_399_071A | 39461369700 | 38098213500 | 96.55 | 0.03 | 97.95 | 93.44 | 43.14 |
| AM549_01R0001 | 14970000000 | 14770000000 | 98.62 | 0.03 | 97.92 | 94.2 | 40.63 |
| AM549_01R0002 | 15250000000 | 15030000000 | 98.61 | 0.02 | 98.13 | 94.7 | 41.14 |
| AM549_01R0003 | 17360000000 | 17150000000 | 98.78 | 0.02 | 98.19 | 94.81 | 40.97 |
| AM549_01R0004 | 17940000000 | 17730000000 | 98.78 | 0.02 | 98.11 | 94.63 | 41.21 |
| AM549_01R0005 | 18350000000 | 18130000000 | 98.76 | 0.02 | 98.19 | 94.81 | 41.18 |
| AM549_01R0006 | 16540000000 | 16350000000 | 98.86 | 0.02 | 98.15 | 94.7 | 41.39 |
| AM549_01R0007 | 15430000000 | 15240000000 | 98.8 | 0.02 | 98.2 | 94.82 | 41.24 |
| AM549_01R0008 | 16400000000 | 16130000000 | 98.31 | 0.02 | 98.18 | 94.79 | 41.41 |
| AM549_01R0009 | 15090000000 | 14880000000 | 98.6 | 0.02 | 98.17 | 94.76 | 40.99 |
| AM549_01R0010 | 15280000000 | 15080000000 | 98.73 | 0.02 | 98.17 | 94.8 | 41.18 |
| AM549_01R0011 | 16380000000 | 16180000000 | 98.77 | 0.02 | 98.28 | 95.01 | 41.08 |
| AM549_01R0012 | 16670000000 | 16470000000 | 98.82 | 0.02 | 98.17 | 94.74 | 40.75 |
| AM549_01R0013 | 17310000000 | 17110000000 | 98.85 | 0.02 | 98.21 | 94.83 | 40.88 |
| AM549_01R0014 | 16290000000 | 16130000000 | 99 | 0.02 | 98.06 | 94.52 | 40.87 |
| AM549_01R0015 | 18900000000 | 18700000000 | 98.95 | 0.02 | 98.14 | 94.67 | 40.6 |
| AM549_01R0016 | 15430000000 | 15190000000 | 98.45 | 0.02 | 98.12 | 94.64 | 40.82 |
| AM549_01R0017 | 17070000000 | 16830000000 | 98.59 | 0.02 | 98.23 | 94.88 | 40.81 |
| AM549_01R0018 | 16730000000 | 16470000000 | 98.46 | 0.02 | 98.16 | 94.69 | 41.08 |
| AM549_01R0004 | 17940000000 | 17730000000 | 98.78 | 0.02 | 98.11 | 94.63 | 41.21 |
| AM549_01R0005 | 18350000000 | 18130000000 | 98.76 | 0.02 | 98.19 | 94.81 | 41.18 |
| AM549_01R0006 | 16540000000 | 16350000000 | 98.86 | 0.02 | 98.15 | 94.7 | 41.39 |
| AM549_01R0007 | 15430000000 | 15240000000 | 98.8 | 0.02 | 98.2 | 94.82 | 41.24 |
| AM549_01R0008 | 16400000000 | 16130000000 | 98.31 | 0.02 | 98.18 | 94.79 | 41.41 |
| AM549_01R0009 | 15090000000 | 14880000000 | 98.6 | 0.02 | 98.17 | 94.76 | 40.99 |
| AM549_01R0010 | 15280000000 | 15080000000 | 98.73 | 0.02 | 98.17 | 94.8 | 41.18 |
| AM549_01R0011 | 16380000000 | 16180000000 | 98.77 | 0.02 | 98.28 | 95.01 | 41.08 |
| AM549_01R0012 | 16670000000 | 16470000000 | 98.82 | 0.02 | 98.17 | 94.74 | 40.75 |
| AM549_01R0013 | 17310000000 | 17110000000 | 98.85 | 0.02 | 98.21 | 94.83 | 40.88 |
| AM549_01R0014 | 16290000000 | 16130000000 | 99 | 0.02 | 98.06 | 94.52 | 40.87 |
| AM549_01R0015 | 18900000000 | 18700000000 | 98.95 | 0.02 | 98.14 | 94.67 | 40.6 |
| AM549_01R0016 | 15430000000 | 15190000000 | 98.45 | 0.02 | 98.12 | 94.64 | 40.82 |
| AM549_01R0017 | 17070000000 | 16830000000 | 98.59 | 0.02 | 98.23 | 94.88 | 40.81 |
| AM549_01R0018 | 16730000000 | 16470000000 | 98.46 | 0.02 | 98.16 | 94.69 | 41.08 |
| AM549_01R0019 | 16210000000 | 15980000000 | 98.58 | 0.02 | 98.16 | 94.73 | 40.73 |
| AM549_01R0020 | 17270000000 | 17040000000 | 98.66 | 0.02 | 98.16 | 94.73 | 40.74 |
| AM549_01R0021 | 14050000000 | 13880000000 | 98.79 | 0.02 | 98.27 | 94.98 | 40.98 |
| AM549_01R0022 | 17170000000 | 17000000000 | 99.04 | 0.02 | 98.28 | 95 | 40.67 |
| AM549_01R0023 | 15830000000 | 15630000000 | 98.73 | 0.02 | 98.16 | 94.78 | 41.3 |
| AM549_01R0024 | 14860000000 | 14720000000 | 99.05 | 0.02 | 98.24 | 94.92 | 40.74 |
| AM549_01R0025 | 15100000000 | 14910000000 | 98.74 | 0.02 | 98.26 | 94.99 | 41.33 |
| AM549_01R0026 | 16250000000 | 16020000000 | 98.58 | 0.02 | 98.32 | 95.08 | 41.57 |
| AM549_01R0027 | 16520000000 | 16310000000 | 98.74 | 0.02 | 98.24 | 94.91 | 41.41 |
| AM549_01R0028 | 15870000000 | 15680000000 | 98.84 | 0.02 | 98.28 | 95.01 | 40.96 |
| AM549_01R0029 | 15160000000 | 14990000000 | 98.92 | 0.02 | 98.26 | 94.93 | 40.96 |
| AM549_01R0030 | 15910000000 | 15750000000 | 98.97 | 0.02 | 98.31 | 95.04 | 40.46 |
| AM549_01R0031 | 17530000000 | 17340000000 | 98.92 | 0.02 | 98.14 | 94.66 | 40.57 |
| AM549_01R0032 | 16740000000 | 16570000000 | 98.97 | 0.02 | 98.23 | 94.82 | 40.38 |
| AM549_01R0033 | 18200000000 | 18010000000 | 98.99 | 0.02 | 98.37 | 95.18 | 40.4 |
| AM549_01R0034 | 15790000000 | 15620000000 | 98.89 | 0.02 | 98.24 | 94.89 | 41.02 |
| AM549_01R0035 | 15080000000 | 14910000000 | 98.82 | 0.02 | 98.28 | 94.96 | 40.67 |
| AM549_01R0036 | 17360000000 | 17140000000 | 98.77 | 0.02 | 98.26 | 94.95 | 41 |
| AM549_01R0037 | 20360000000 | 20130000000 | 98.89 | 0.02 | 98.31 | 95.05 | 40.65 |
| AM549_01R0038 | 16840000000 | 16640000000 | 98.83 | 0.02 | 98.22 | 94.88 | 40.91 |
| AM549_01R0039 | 17210000000 | 17020000000 | 98.9 | 0.02 | 98.28 | 95.02 | 40.97 |
| AM549_01R0040 | 15430000000 | 15280000000 | 99.09 | 0.03 | 97.63 | 93.52 | 40.22 |
| AM549_01R0041 | 14560000000 | 14380000000 | 98.77 | 0.02 | 98 | 94.38 | 40.5 |
| AM549_01R0042 | 17270000000 | 16800000000 | 97.3 | 0.03 | 97.79 | 93.73 | 42.3 |
| AM549_01R0043 | 16800000000 | 16390000000 | 97.56 | 0.03 | 97.67 | 93.42 | 41.9 |
| AM549_01R0044 | 16970000000 | 16550000000 | 97.55 | 0.03 | 97.74 | 93.62 | 42.6 |
| AM549_01R0045 | 13960000000 | 13640000000 | 97.68 | 0.03 | 97.83 | 93.8 | 41.81 |
| AM549_01R0046 | 16230000000 | 15850000000 | 97.61 | 0.03 | 97.91 | 94.04 | 42.92 |
| AM549_01R0047 | 15820000000 | 15430000000 | 97.55 | 0.03 | 97.84 | 93.85 | 43.06 |
| AM549_01R0048 | 17320000000 | 16900000000 | 97.57 | 0.03 | 97.86 | 93.97 | 42.66 |
| AM549_01R0049 | 14450000000 | 13980000000 | 96.74 | 0.03 | 97.93 | 94.11 | 43.06 |
| AM549_01R0050 | 17030000000 | 16740000000 | 98.27 | 0.03 | 97.89 | 94 | 42.68 |
| AM549_01R0051 | 15990000000 | 15770000000 | 98.59 | 0.03 | 97.94 | 94.09 | 41.81 |
| AM549_01R0052 | 15100000000 | 14840000000 | 98.28 | 0.03 | 97.84 | 93.93 | 43.15 |
| AM549_01R0053 | 16720000000 | 16380000000 | 97.95 | 0.03 | 97.87 | 93.98 | 42.95 |
| AM549_01R0054 | 16630000000 | 16400000000 | 98.65 | 0.03 | 97.69 | 93.39 | 41.37 |
| AM549_01R0055 | 16620000000 | 16370000000 | 98.54 | 0.03 | 97.76 | 93.59 | 41.08 |
| AM549_01R0056 | 16850000000 | 16580000000 | 98.41 | 0.03 | 97.9 | 93.96 | 41.41 |
| AM549_01R0057 | 17080000000 | 16710000000 | 97.8 | 0.03 | 97.76 | 93.66 | 42.07 |
| AM549_01R0058 | 15170000000 | 14900000000 | 98.25 | 0.02 | 98.11 | 94.4 | 41.21 |
| AM549_01R0059 | 18890000000 | 18620000000 | 98.54 | 0.03 | 97.77 | 93.58 | 40.77 |

**Table S3** Sequencing depth and coverage statistics

| Sample | Clean_reads | mapped_reads | mapping_rate | Average_depth | Coverage_1X | Coverage_4X |
| --- | --- | --- | --- | --- | --- | --- |
| C_112A | 285194000 | 279352907 | 97.95% | 14.67 | 96.11% | 91.48% |
| C_13A | 234892206 | 229550629 | 97.73% | 12.38 | 95.94% | 89.35% |
| C_23A | 221009030 | 216182347 | 97.82% | 11.69 | 95.88% | 88.50% |
| C_34 | 242352694 | 236990703 | 97.79% | 12.60 | 96.03% | 89.49% |
| C_43A | 365016462 | 356731550 | 97.73% | 18.09 | 96.45% | 92.47% |
| C_4A | 219339176 | 214929730 | 97.99% | 11.60 | 95.81% | 88.33% |
| C_51A | 177934804 | 173983393 | 97.78% | 9.79 | 95.32% | 84.74% |
| C_54A | 265454152 | 259568590 | 97.78% | 13.76 | 96.04% | 90.97% |
| C_57A | 214638570 | 210116382 | 97.89% | 11.43 | 95.68% | 88.82% |
| C_59A | 243082226 | 237763882 | 97.81% | 12.83 | 95.90% | 89.52% |
| C_61A | 246099680 | 240831522 | 97.86% | 12.77 | 95.82% | 90.24% |
| C_62A | 186387612 | 181326580 | 97.28% | 10.09 | 95.35% | 85.37% |
| C_63A | 230883624 | 225708981 | 97.76% | 12.08 | 95.70% | 89.64% |
| C_69A | 513876628 | 502767588 | 97.84% | 25.42 | 97.06% | 94.26% |
| C_6A | 239936170 | 234879403 | 97.89% | 12.65 | 96.06% | 89.61% |
| C_71A | 224773410 | 220037691 | 97.89% | 11.87 | 95.78% | 89.52% |
| C_86A | 223058840 | 217731564 | 97.61% | 11.78 | 95.87% | 88.60% |
| C_95B | 328270670 | 321430820 | 97.92% | 16.44 | 96.53% | 92.15% |
| J_03YAOJIA | 259209394 | 253845545 | 97.93% | 13.52 | 96.14% | 90.46% |
| J_10GUJIA | 244423420 | 239232800 | 97.88% | 12.79 | 95.88% | 90.25% |
| J_10YAOJIA | 229169812 | 224303453 | 97.88% | 12.04 | 95.75% | 89.57% |
| J_190A | 257774700 | 252255482 | 97.86% | 13.51 | 95.89% | 90.82% |
| J_1HYAOGUA | 256336426 | 250411199 | 97.69% | 13.27 | 96.12% | 90.24% |
| J_209A | 253356232 | 248021746 | 97.89% | 13.24 | 96.07% | 90.23% |
| J_2GUJIA | 256585612 | 251300088 | 97.94% | 13.21 | 96.09% | 90.19% |
| J_3GUJIA | 210091960 | 205770178 | 97.94% | 11.35 | 95.76% | 88.10% |
| J_4YAOJIA | 249932788 | 244870092 | 97.97% | 13.05 | 96.04% | 90.07% |
| J_67A | 247995472 | 243005685 | 97.99% | 12.86 | 95.86% | 90.24% |
| J_9GUJIA | 276215664 | 270521867 | 97.94% | 14.18 | 95.98% | 91.19% |
| J_9YAOJIA | 255448208 | 250185371 | 97.94% | 13.26 | 95.90% | 90.62% |
| J_B08YAOJIA | 259252236 | 253943811 | 97.95% | 13.35 | 95.88% | 90.70% |
| J_B43TUIJIA | 294371120 | 288327652 | 97.95% | 15.11 | 96.11% | 91.63% |
| M_100019A | 176465604 | 173034333 | 98.06% | 9.28 | 95.00% | 82.02% |
| M_100073A | 271665144 | 266268619 | 98.01% | 13.95 | 96.04% | 91.29% |
| M_10_GUJIA | 216019764 | 211354594 | 97.84% | 11.41 | 95.67% | 88.07% |
| M_110002A | 196408396 | 192200017 | 97.86% | 10.53 | 95.23% | 86.56% |
| M_110008A | 243868312 | 239249158 | 98.11% | 12.16 | 96.07% | 89.64% |
| M_110020A | 249480002 | 244694052 | 98.08% | 12.32 | 96.07% | 89.77% |
| M_11_YANJIA | 257960732 | 252185418 | 97.76% | 13.37 | 96.07% | 90.28% |
| M_120023A | 173973170 | 170730054 | 98.14% | 9.25 | 94.93% | 81.70% |
| M_120038A | 192280250 | 188624212 | 98.10% | 10.06 | 95.29% | 85.23% |
| M_12_YAOJIA | 270430732 | 264774735 | 97.91% | 13.92 | 96.14% | 90.66% |
| M_130006A | 173110220 | 169870910 | 98.13% | 9.22 | 94.85% | 80.96% |
| M_130032A | 252381286 | 247677692 | 98.14% | 12.08 | 95.78% | 88.67% |
| M_130033A | 245513250 | 240991516 | 98.16% | 12.13 | 95.95% | 89.55% |
| M_130034A | 231691066 | 227222979 | 98.07% | 11.57 | 95.79% | 88.37% |
| M_13_YANJIA | 327922946 | 320981558 | 97.88% | 16.59 | 96.49% | 92.13% |
| M_140005A | 178818398 | 175218216 | 97.99% | 9.39 | 95.12% | 82.78% |
| M_140007A | 255637718 | 250266078 | 97.90% | 12.49 | 96.14% | 90.16% |
| M_140009_4_A | 180576596 | 177155633 | 98.11% | 9.52 | 95.11% | 82.82% |
| M_140026A | 265651524 | 260510505 | 98.06% | 12.97 | 96.19% | 90.49% |
| M_140032A | 279192396 | 273241705 | 97.87% | 13.75 | 96.33% | 91.24% |
| M_140047A | 189196386 | 185373787 | 97.98% | 10.06 | 95.36% | 85.58% |
| M_140056A | 181731822 | 178255682 | 98.09% | 9.58 | 95.11% | 83.34% |
| M_150008A | 202565908 | 198541024 | 98.01% | 10.53 | 95.41% | 86.44% |
| M_150010A | 181692838 | 178199624 | 98.08% | 9.62 | 95.19% | 83.86% |
| M_150024_6_A | 207394350 | 203186494 | 97.97% | 11.02 | 95.40% | 87.88% |
| M_150073A | 308528290 | 302599463 | 98.08% | 15.18 | 96.32% | 91.63% |
| M_1596A | 208369064 | 204523903 | 98.15% | 10.62 | 95.52% | 86.50% |
| M_15_GUJIA | 254280082 | 249107913 | 97.97% | 13.10 | 96.03% | 90.00% |
| M_160001A | 258716560 | 253574846 | 98.01% | 12.95 | 96.02% | 90.18% |
| M_160006A | 262422948 | 257343065 | 98.06% | 12.95 | 96.17% | 90.51% |
| M_160011_11_A | 253070994 | 248299938 | 98.11% | 12.40 | 96.00% | 89.63% |
| M_160012A | 175682192 | 172275235 | 98.06% | 9.30 | 95.13% | 82.56% |
| M_160013A | 242751082 | 238226317 | 98.14% | 12.15 | 95.90% | 89.20% |
| M_160015A | 195966810 | 192120319 | 98.04% | 10.16 | 95.36% | 85.69% |
| M_160023A | 240386862 | 235893240 | 98.13% | 11.89 | 95.88% | 88.87% |
| M_160039A | 203525828 | 199289759 | 97.92% | 10.88 | 95.37% | 87.49% |
| M_16_YANJIA | 312145670 | 305761076 | 97.95% | 15.86 | 96.35% | 91.83% |
| M_170007A | 298342030 | 292291284 | 97.97% | 14.76 | 95.79% | 90.74% |
| M_170014_12_A | 171308070 | 168068388 | 98.11% | 9.12 | 94.84% | 81.17% |
| M_180001A | 261079106 | 256089124 | 98.09% | 12.87 | 95.92% | 89.79% |
| M_180036A | 206969178 | 202975569 | 98.07% | 10.79 | 95.57% | 87.21% |
| M_180037A | 243784248 | 238901069 | 98.00% | 12.47 | 95.88% | 89.68% |
| M_180054A | 204533206 | 200517273 | 98.04% | 10.50 | 95.53% | 86.44% |
| M_180058A | 280825492 | 275533990 | 98.12% | 13.48 | 96.21% | 90.73% |
| M_180073A | 238693436 | 234345981 | 98.18% | 11.63 | 95.82% | 88.55% |
| M_190063A | 242666444 | 238056149 | 98.10% | 11.91 | 95.98% | 89.22% |
| M_19_YANJIA | 281886660 | 276184545 | 97.98% | 14.46 | 96.27% | 91.06% |
| M_1_YANJIA | 274963804 | 269054042 | 97.85% | 14.07 | 96.12% | 90.79% |
| M_2_YANJIA | 233381636 | 228545764 | 97.93% | 12.28 | 95.89% | 89.03% |
| M_3-YAOJIA | 248482320 | 243135207 | 97.85% | 12.93 | 95.97% | 89.79% |
| M_51896A | 487975690 | 478850754 | 98.13% | 22.68 | 97.10% | 94.11% |
| M_5_GUJIA | 249364290 | 244349060 | 97.99% | 12.84 | 95.85% | 89.53% |
| M_5_YANJIA | 287011066 | 281159770 | 97.96% | 14.62 | 96.26% | 91.19% |
| M_6_YAOJIA | 248634098 | 243422126 | 97.90% | 13.02 | 96.00% | 89.86% |
| M_788427A | 237305560 | 231894551 | 97.72% | 11.62 | 95.93% | 88.75% |
| M_7_YAOJIA | 284765136 | 278726206 | 97.88% | 14.54 | 96.29% | 91.16% |
| M_8_YAOJIA | 235607580 | 230501851 | 97.83% | 12.34 | 95.93% | 89.33% |
| M_9_YAOJIA | 229134558 | 224525736 | 97.99% | 12.03 | 95.66% | 88.53% |
| M_A1361YA | 213238772 | 208869207 | 97.95% | 11.06 | 95.68% | 88.25% |
| M_A1508A | 236864988 | 232167493 | 98.02% | 11.79 | 95.81% | 88.64% |
| M_C160001A | 406681662 | 397980889 | 97.86% | 19.86 | 96.45% | 93.18% |
| M_C170004A | 182765420 | 178039012 | 97.41% | 9.73 | 95.12% | 84.49% |
| M_D024A | 260154966 | 251510647 | 96.68% | 13.17 | 96.08% | 90.88% |
| M_D045A | 224056500 | 218291048 | 97.43% | 11.14 | 95.81% | 88.09% |
| M_D072A | 277147894 | 270934185 | 97.76% | 13.64 | 96.24% | 91.05% |
| M_D401_2118A | 200789844 | 197007826 | 98.12% | 10.36 | 95.48% | 85.77% |
| M_F1895A | 259442078 | 254363112 | 98.04% | 12.86 | 96.20% | 90.48% |
| M_M190007 | 220920330 | 216616721 | 98.05% | 11.05 | 95.65% | 87.34% |
| M_Q1882A | 182383304 | 178976307 | 98.13% | 9.48 | 95.08% | 82.59% |
| MU_01A | 270804244 | 265283938 | 97.96% | 14.05 | 96.01% | 91.10% |
| MU_02A | 269807476 | 264308937 | 97.96% | 14.06 | 95.98% | 91.14% |
| MU_03B | 245387186 | 240612959 | 98.05% | 12.59 | 95.83% | 90.17% |
| MU_04B | 280903152 | 275445545 | 98.06% | 14.31 | 96.00% | 91.32% |
| MU_05A | 362004742 | 354814951 | 98.01% | 18.02 | 96.02% | 92.39% |
| MU_06A | 239573932 | 234725286 | 97.98% | 12.62 | 95.80% | 90.12% |
| MU_08A | 231003108 | 226387860 | 98.00% | 12.15 | 95.70% | 89.70% |
| MU_09A | 253838718 | 248775276 | 98.01% | 13.27 | 95.92% | 90.66% |
| MU_10A | 219835078 | 215392538 | 97.98% | 11.69 | 95.54% | 89.14% |
| MU_11A | 294360112 | 288373953 | 97.97% | 15.23 | 96.19% | 91.73% |
| MU_12A | 231108932 | 226462548 | 97.99% | 12.20 | 95.76% | 89.73% |
| MU_13B | 220503152 | 216156010 | 98.03% | 11.62 | 95.66% | 89.21% |
| MU_14A | 239600870 | 234799300 | 98.00% | 12.56 | 95.82% | 90.11% |
| MU_16B | 307947064 | 302024470 | 98.08% | 15.43 | 96.42% | 91.63% |
| MU_17A | 248378626 | 243381664 | 97.99% | 13.00 | 95.82% | 90.44% |
| MU_18A | 246864598 | 241879773 | 97.98% | 13.11 | 96.05% | 90.01% |
| MU_19A | 243896148 | 239048221 | 98.01% | 12.89 | 95.79% | 89.55% |
| MU_20A | 229049348 | 224370100 | 97.96% | 12.21 | 95.85% | 89.01% |
| Z_002A | 215713500 | 211662038 | 98.12% | 11.12 | 95.58% | 87.70% |
| Z_005_0095A | 186803112 | 183509873 | 98.24% | 9.61 | 95.05% | 82.68% |
| Z_0057A | 238034812 | 233579673 | 98.13% | 11.99 | 95.79% | 88.84% |
| Z_0065A | 199007820 | 195285425 | 98.13% | 10.24 | 95.62% | 84.71% |
| Z_0078_041A | 212150400 | 207935051 | 98.01% | 11.23 | 95.53% | 88.06% |
| Z_0080_012A | 197544140 | 193729590 | 98.07% | 10.28 | 95.54% | 86.62% |
| Z_0094_007A | 201694148 | 197976791 | 98.16% | 10.17 | 95.42% | 85.18% |
| Z_021A | 204391702 | 200540133 | 98.12% | 10.39 | 95.59% | 86.43% |
| Z_024A | 188045628 | 184778608 | 98.26% | 9.68 | 95.11% | 83.02% |
| Z_031_374A | 226390282 | 221893879 | 98.01% | 11.87 | 96.00% | 89.05% |
| Z_039_040A | 217965622 | 213996442 | 98.18% | 10.81 | 95.59% | 86.56% |
| Z_318A | 232293520 | 228117832 | 98.20% | 11.49 | 95.71% | 88.14% |
| Z_321A | 206581072 | 202875104 | 98.21% | 10.54 | 95.43% | 85.95% |
| Z_340A | 286797214 | 281279938 | 98.08% | 14.07 | 96.31% | 91.26% |
| Z_353_1A | 227278056 | 222765024 | 98.01% | 11.67 | 95.73% | 88.95% |
| Z_353_2A | 210552988 | 206665266 | 98.15% | 10.81 | 95.54% | 87.03% |
| Z_374A | 225122048 | 221124722 | 98.22% | 11.13 | 95.65% | 87.25% |
| Z_378A | 249473690 | 245084993 | 98.24% | 12.19 | 95.88% | 89.14% |
| Z_389A | 243630084 | 239018596 | 98.11% | 12.00 | 96.01% | 89.29% |
| Z_399_071A | 253988090 | 249418836 | 98.20% | 12.45 | 96.10% | 88.93% |
| AM549_01R0001 | 98447974 | 96011452 | 97.53% | 3.74 | 86.08% | 34.2% |
| AM549_01R0002 | 100225986 | 97841251 | 97.62% | 3.62 | 86.06% | 31.74% |
| AM549_01R0003 | 114315888 | 111570731 | 97.6% | 3.9 | 87.76% | 37.17% |
| AM549_01R0004 | 118168888 | 115418028 | 97.67% | 4.01 | 88.19% | 38.56% |
| AM549_01R0005 | 120837662 | 118049859 | 97.69% | 4 | 88.26% | 38.46% |
| AM549_01R0006 | 108991568 | 106459835 | 97.68% | 3.85 | 87.76% | 36.13% |
| AM549_01R0007 | 101623536 | 99233213 | 97.65% | 3.65 | 86.11% | 32% |
| AM549_01R0008 | 107512726 | 105027650 | 97.69% | 3.75 | 86.94% | 33.92% |
| AM549_01R0009 | 99185742 | 96847770 | 97.64% | 3.62 | 85.37% | 31.24% |
| AM549_01R0010 | 100541804 | 98171969 | 97.64% | 3.58 | 86.13% | 31.41% |
| AM549_01R0011 | 107834756 | 105283983 | 97.63% | 3.82 | 87.01% | 35.32% |
| AM549_01R0012 | 109815302 | 107153703 | 97.58% | 3.92 | 87.62% | 37.59% |
| AM549_01R0013 | 114058220 | 111371836 | 97.64% | 3.87 | 87.45% | 36.54% |
| AM549_01R0014 | 107540698 | 104904217 | 97.55% | 3.75 | 86.97% | 34.41% |
| AM549_01R0015 | 124659286 | 121615512 | 97.56% | 4.3 | 88.65% | 42.19% |
| AM549_01R0016 | 101246858 | 98827406 | 97.61% | 3.68 | 85.95% | 32.56% |
| AM549_01R0017 | 112168522 | 109514636 | 97.63% | 3.91 | 86.91% | 36.41% |
| AM549_01R0018 | 109826468 | 107277594 | 97.68% | 3.83 | 87.11% | 35.56% |
| AM549_01R0019 | 106500144 | 103945064 | 97.6% | 3.73 | 86.26% | 33.68% |
| AM549_01R0020 | 113612248 | 110879823 | 97.59% | 3.92 | 87.38% | 37.07% |
| AM549_01R0021 | 92539848 | 90323112 | 97.6% | 3.45 | 84.74% | 29.05% |
| AM549_01R0022 | 113346224 | 110617636 | 97.59% | 3.96 | 88.09% | 39.15% |
| AM549_01R0023 | 104221598 | 101796308 | 97.67% | 3.71 | 86.65% | 33.13% |
| AM549_01R0024 | 98129106 | 95815292 | 97.64% | 3.6 | 85.28% | 31.04% |
| AM549_01R0025 | 99412562 | 97097462 | 97.67% | 3.63 | 86.03% | 32.16% |
| AM549_01R0026 | 106791896 | 104356738 | 97.72% | 3.73 | 87.23% | 34.14% |
| AM549_01R0027 | 108730810 | 106189277 | 97.66% | 3.75 | 87.59% | 35.38% |
| AM549_01R0028 | 104551252 | 102088666 | 97.64% | 3.69 | 86.56% | 33.97% |
| AM549_01R0029 | 99953910 | 97329833 | 97.37% | 3.6 | 85.75% | 32.13% |
| AM549_01R0030 | 104988640 | 102457509 | 97.59% | 3.74 | 86.26% | 34.71% |
| AM549_01R0031 | 115609454 | 112679211 | 97.47% | 4.02 | 87.46% | 38.42% |
| AM549_01R0032 | 110460302 | 107777628 | 97.57% | 3.82 | 86.49% | 35.3% |
| AM549_01R0033 | 120087582 | 117195684 | 97.59% | 3.98 | 87.48% | 38.73% |
| AM549_01R0034 | 104116054 | 101651539 | 97.63% | 3.68 | 86.21% | 32.92% |
| AM549_01R0035 | 99373708 | 96980504 | 97.59% | 3.62 | 85.34% | 32.05% |
| AM549_01R0036 | 114287826 | 111608684 | 97.66% | 3.89 | 87.21% | 36.39% |
| AM549_01R0037 | 134226766 | 130947046 | 97.56% | 4.26 | 89.35% | 44.12% |
| AM549_01R0038 | 110961210 | 108352810 | 97.65% | 3.91 | 87.02% | 36.15% |
| AM549_01R0039 | 113469630 | 110769303 | 97.62% | 3.85 | 87.49% | 36.26% |
| AM549_01R0040 | 101899882 | 99291696 | 97.44% | 3.79 | 87.31% | 36.99% |
| AM549_01R0041 | 95878370 | 93498090 | 97.52% | 3.59 | 85.34% | 31.93% |
| AM549_01R0042 | 112005586 | 109252478 | 97.54% | 4.02 | 87.84% | 38.42% |
| AM549_01R0043 | 109285198 | 106616499 | 97.56% | 3.97 | 87.53% | 37.82% |
| AM549_01R0044 | 110333612 | 107702947 | 97.62% | 4.03 | 87.76% | 38.28% |
| AM549_01R0045 | 90920494 | 88715561 | 97.57% | 3.49 | 84.46% | 29.77% |
| AM549_01R0046 | 105638228 | 103338148 | 97.82% | 3.91 | 86.93% | 35.57% |
| AM549_01R0047 | 102879526 | 100642811 | 97.83% | 3.84 | 86.66% | 34.47% |
| AM549_01R0048 | 112660294 | 110128074 | 97.75% | 4.11 | 87.73% | 38.47% |
| AM549_01R0049 | 93219164 | 91173773 | 97.81% | 3.63 | 84.36% | 30.04% |
| AM549_01R0050 | 111591588 | 109090226 | 97.76% | 3.94 | 87.54% | 36.98% |
| AM549_01R0051 | 105113840 | 102686646 | 97.69% | 3.85 | 87.35% | 36.7% |
| AM549_01R0052 | 98911356 | 96727584 | 97.79% | 3.73 | 86.32% | 32.39% |
| AM549_01R0053 | 109213744 | 106806633 | 97.8% | 3.97 | 87.22% | 36.11% |
| AM549_01R0054 | 109365470 | 106753741 | 97.61% | 4 | 87.14% | 38.49% |
| AM549_01R0055 | 109150910 | 106519998 | 97.59% | 3.99 | 86.85% | 38.47% |
| AM549_01R0056 | 110565574 | 107940786 | 97.63% | 3.99 | 87.08% | 38.6% |
| AM549_01R0057 | 111395546 | 108795355 | 97.67% | 4.02 | 87.57% | 38.43% |
| AM549_01R0058 | 99343802 | 97004046 | 97.64% | 3.79 | 86.37% | 35.81% |
| AM549_01R0059 | 124114050 | 121118161 | 97.59% | 4.31 | 88.61% | 45% |

**Table S4** Genetic diversity correlation index sizes of six yak breed/population

| Breeds/Population | Pi | *Ho* | *He* | F |
| --- | --- | --- | --- | --- |
| Maiwa yak (MW) | 0.00145 | 0.29861 | 0.30402 | 0.01778 |
| Muli yak (ML) | 0.00129 | 0.36546 | 0.33848 | -0.07969 |
| Jinchuan yak (JC) | 0.00149 | 0.32407 | 0.32323 | -0.00260 |
| Changtai yak (CT) | 0.00153 | 0.31438 | 0.30747 | -0.02247 |
| Zhongdian yak (ZD) | 0.00138 | 0.29013 | 0.29477 | 0.01577 |
| Tibetan yak (XZ) | 0.00104 | 0.27363 | 0.29020 | 0.05707 |

Pi: nucleotide diversity. *Ho*: observed heterozygosity. *He*: expected heterozygosity. *F*: inbreeding coefficient.
